# Phase Transition Behavior and Catalytic Activity of Poly(*N*-acryloylglycinamide-*co*-methacrylic acid) Microgels

**DOI:** 10.1021/acs.langmuir.0c03264

**Published:** 2021-02-17

**Authors:** Dong Yang, Heli Eronen, Heikki Tenhu, Sami Hietala

**Affiliations:** Department of Chemistry, University of Helsinki, P.O. Box 55, FIN-00014 HU Helsinki, Finland

## Abstract

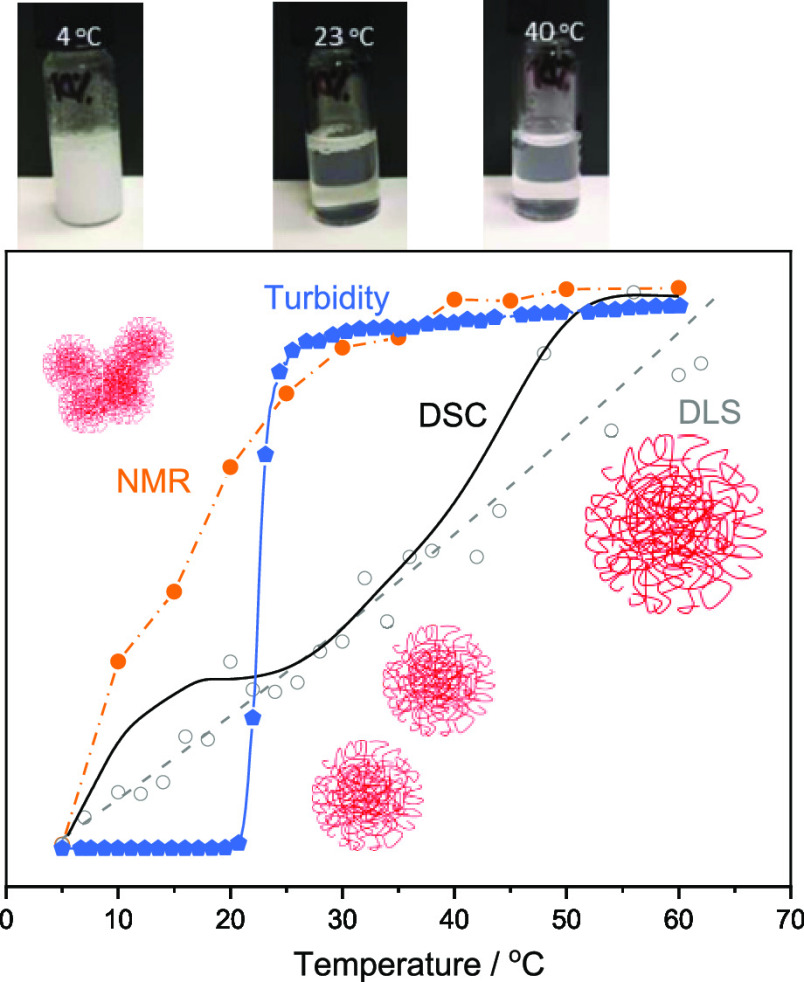

Poly(*N*-acryloyl glycinamide) is a well-known thermoresponsive polymer possessing an upper critical solution temperature (UCST) in water. By copolymerizing *N*-acryloyl glycinamide (NAGA) with methacrylic acid (MAA) in the presence of a crosslinker, poly(*N*-acryloyl glycinamide-*co*-methacrylic acid) [P(NAGA–MAA)] copolymer microgels with an MAA molar fraction of 10–70 mol % were obtained. The polymerization kinetics suggests that the copolymer microgels have a random structure. The size of the microgels was between 60 and 120 nm in the non-aggregated swollen state in aqueous medium and depending on the solvent conditions, they show reversible swelling and shrinking upon temperature change. Their phase transition behavior was studied by a combination of methods to understand the process of the UCST-type behavior and interactions between NAGA and MAA. P(NAGA–MAA) microgels were loaded with silver nanoparticles (AgNPs) by the reduction of AgNO_3_ under UV light. Compared with the chemical reduction of AgNO_3_, the photoreduction results in smaller AgNPs and the amount and size of the AgNPs are dependent on the comonomer ratio. The catalytic activity of the AgNP-loaded microgels in 4-nitrophenol reduction was tested.

## Introduction

Responsive microgels^1,2^ loaded with inorganic nanoparticles have gained much research interest for catalysis applications.^3−14^ These hybrid microgels display enhanced catalytic performance, for example, due to the improved stability of the nanoparticles within the polymer networks, tunability of the catalytic properties, and recyclability of the catalyst by utilizing the stimuli-responsivity of the microgels. Most studies are concentrated on the use of lower critical solution-type (LCST) microgels, especially poly(*N*-isopropyl acrylamide) (PNIPAM). These materials undergo a volume phase transition, contraction, or collapse upon heating in aqueous dispersions. This behavior is retained after the addition of catalytically active components, for example, enzymes or noble metal nanoparticles. When aiming at a more efficient encapsulation of metal nanoparticles inside the microgels, copolymerization of a negatively charged comonomer with a thermally responsive monomer has been used.^15−17^ The charged units, most often acrylic acid, enable increased interactions of the metal cations within the crosslinked polymer structure prior to reduction and lead to higher nanoparticle content.^17−20^ However, the copolymer phase transition temperature may change with the monomer ratio. With the LCST-type systems such as PNIPAM, the charged, hydrophilic component often increases the phase transition temperature, but the effect is not straightforward.^21,22^ An important aspect regarding catalysis applications is that the LCST-type behavior can be considered a disadvantage because the diffusion of reactants inside the microgel particles is compromised upon heating, leading to lower catalytic activity around the phase transition.^4,23,24^

Upper critical solution temperature (UCST)-type polymers are tempting due to their opposite thermal behavior—upon increase of temperature, the polymer dissolves.^25^ When a crosslinker is used as in the case of microgels, dissolution is not possible, but the polymer networks swell. This in turn allows faster diffusion of the reactants to the nanoreactors and enables higher turnover in the catalysis. Catalytic UCST-type microgels have been reported^23,26,27^ mostly based on poly(*N*-acryloyl glycinamide) [P(NAGA)].^28^ It has been shown that these microgels show increased catalytic activity compared to their LCST counterparts and furthermore the catalytic activity can be suppressed by cooling the system.^26^

The approach of using negatively charged comonomers to increase the catalyst loading similar to, for example, PNIPAM has drawbacks in the case of P(NAGA). Its UCST-type phase transition behavior is based on delicate interactions between the monomer units and even minute amounts of acrylic acid residues have been shown to suppress the phase transition behavior.^29^ However, linear poly(*N*-acryloyl glycinamide-*co*-methacrylic acid) [P(NAGA–MAA)] copolymers have been found to have a higher phase transition temperature compared with P(NAGA) homopolymers.^30^ Compared to poly(acrylic acid), PAA, P(MAA) is more hydrophobic and shows a stronger complexation with hydrogen bond acceptor polymers due to the additional methyl group on the polymer backbone.^31^ This occurs *via* intra- and intermolecular hydrogen bonding interactions between the monomer units. These interactions are however pH-dependent and strengthen in acidic conditions below the p*K*_a_ of MAA. This pH dependence adds another dimension for the application of these thermoresponsive systems.

In the present study, we synthesized P(NAGA–MAA) microgels and then loaded them with photocatalytically reduced AgNPs. The phase transition behavior of the microgels with different ratios of NAGA and MAA was investigated, followed by the study of their catalyst encapsulation capacity and catalytic efficiency.

## Experimental Section

### Materials

Glycinamide hydrochloride (Bachem), potassium carbonate (Fisher Scientific), acryloyl chloride (Sigma-Aldrich), anhydrous diethyl ether (J.T. Baker), acetone, and methanol (both from Sigma-Aldrich) were all used as received to prepare the NAGA monomer according to the procedure described in the literature.^26^*N*,*N*,*N*′,*N*′-tetramethylethylenediamine (TEMED) and sodium dodecyl sulfate (SDS) (both from Sigma) were used as received. Ammonium persulfate (APS) and *N*,*N*′-methylenebis(acrylamide) (BIS) were recrystallized, and MAA was distilled before use. Other substances and solvents with the highest purity were used as received. Deionized water was used in the syntheses and in the dialysis. For the dialysis, the membranes had a molecular weight cutoff of 12–14 kDa.

### Synthesis of P(NAGA–MAA) Microgels

The microgels were prepared using slightly modified conditions of synthesis of P(NAGA) microgels reported earlier.^26^ For all syntheses, the total monomer concentration was 72 mM, including 3 mol % BIS with respect to the monomers, in 25 mL of deionized water. SDS (4 mM) was added and the solution purged with N_2_ for 30 min followed by addition of an APS (6 mM) and TEMED (12 mM) mixture. The homopolymer microgel was prepared at 0 °C in an ice bath, but for the copolymer microgels, the reaction conditions were tuned slightly as MAA has a melting point of 15 °C and it is soluble in water only above the temperature of 16 °C. Hence, the reaction temperature was adjusted to 16 °C, even below the assumed volume phase transition temperature (VPTT) of the polymers according to the literature.^30^ The reactions were allowed to proceed overnight. Polymerization conversions were checked by ^1^H NMR at the beginning and end of the reactions. A linear P(NAGA) homopolymer was prepared as a comparison without added BIS.

The P(NAGA–MAA) copolymer microgel dispersions were first purified by dialysis followed by freeze-drying. Dry microgel powders were then dispersed in methanol and stirred overnight to remove the remaining adsorbed TEMED. The mixture was sonicated for 10 min before collecting the product by centrifugation and removing the methanol layer. The washing procedure was repeated three times. To obtain the microgels, the dried powders were dispersed in deionized water and collected by freeze-drying.

### Copolymerization Kinetics

The kinetics were followed by ^1^H NMR measurements to monitor the reactivity of the two monomers. For NAGA 70 mol % and MAA 30 mol % polymerization, NAGA (6.28 mg, 0.049 mmol), MAA (1.81 mg, 0.021 mmol), BIS (0.32 mg, 2.08 μmol), and SDS (1.15 mg, 3.99 μmol) were added to a 5 mL vial and dissolved in 0.75 mL of D_2_O. The solution was purged with nitrogen for 15 min. After purging, the solution was transferred to a nitrogen-filled NMR tube. The reaction was initiated by addition of nitrogen-purged APS (1.37 mg, 6.00 μmol) and TEMED (1.39 mg, 11.96 μmol) solutions so that the final volume of the reaction solution was 1 mL. The temperature was set to 16 °C and the reaction was followed overnight. The same procedure was used for NAGA 50 mol%–MAA 50 mol % polymerization, adjusting the reactant ratios accordingly.

### AgNP Synthesis

Microgel dispersions (1.5 mL; 10 mg/mL in D_2_O) mixed with AgNO_3_ (1.5 mL; 0.1 M) were subjected to 365 nm UV light with a nominal 36 W output for 2 h at room temperature. The AgNP formation was followed by UV. After the reactions, the samples were dialyzed against distilled water and the UV-spectra measured. For comparison, a chemically reduced AgNP–P(NAGA) microgel was prepared as reported earlier.^26^

### Catalysis Studies

For catalysis studies, the Ag-microgel dispersion (13 μL; 5 mg/mL; deionized water) was added to 4-nitrophenol solution (2.5 mL; 0.01 mg/mL; 0.072 mM) in a 10 mm quartz cuvette containing fresh NaBH_4_ (3.8 mg/mL; 100 mM) at 30 °C. The pH of the reaction medium was 9. The reduction of 4-nitrophenol to 4-aminophenol was followed by UV monitoring the absorbance change at 400 nm.

### Characterization

For the microgel characterization, the concentrations of the samples were 10 mg/mL in D_2_O unless otherwise stated. ^1^H NMR measurements were made using a 500 MHz Bruker AVANCE III spectrometer. For variable temperature measurements, the temperature was increased stepwise from 5 to 60 °C and the spectra were measured 5 °C intervals, allowing the sample to equilibrate for 10 min. Signal intensities were calculated from the respective NAGA and MAA signals.

Transmittance of the dispersions was studied by using a JASCO V-750 UV–visible spectrophotometer with a JASCO CTU-100 circulating thermostat unit. The temperature range was from 5 to 60 °C and the rate was 1 °C/min in both heating and cooling.

Dynamic light scattering (DLS) measurements were made on 1 mg/mL dispersions in deionized water with pH adjusted to pH 3. The measurement setup consisted of a BI-200SM goniometer, a BIC-TurboCorr digital pseudo-cross-correlator, a BI-CrossCorr detector, and a LAUDA RC 6 CP thermostat to control the temperature. The laser was operated at 488 nm observing the scattering at a 90° angle. The hydrodynamic diameters, *D*_h_, were determined using the inbuilt CONTIN-algorithm of Brookhaven software. Additionally, size changes upon heating and cooling scans were determined with a Malvern Zetasizer Nano ZS (laser wavelength: 633 nm, scattering angle: 173°). The effective (*Z*-average) diameters were obtained from the second-order cumulant fit and the size distributions using the Malvern inbuilt multiexponential fit.

Microcalorimetric measurements were performed with Malvern MicroCal PEAQ-DSC equipped with a measuring cell of 0.13 mL. The temperature range was from 5 to 95 °C and the rate was 1 °C/min in both heating and cooling. Thermal scans using the same solvent in both cells as in the sample measurements were used to establish the baselines. All samples were degassed prior to loading the cell.

The pH of the dispersions was determined by a VWR pHenomenal IS 2100L pH-meter calibrated with three standards prior to use.

TEM measurements were carried out using a JEOL JEM-1400 transmission electron microscope. The microgel samples were placed on a Pioloform-coated, glow discharge-treated 200 mesh Cu grids and dried at ambient temperature.

TGA measurements were performed with a NETZSCH Jupiter STA 449 F3. The samples were heated from room temperature to 800 °C under a nitrogen atmosphere at 10 °C/min. The Ag content was calculated by comparing the mass difference between the residues of the microgel without AgNPs and those of the AgNP-loaded ones at 800 °C.

## Results and Discussion

### Synthesis and Stimuli-Responsive Properties of the Microgels

**Figure 1:**
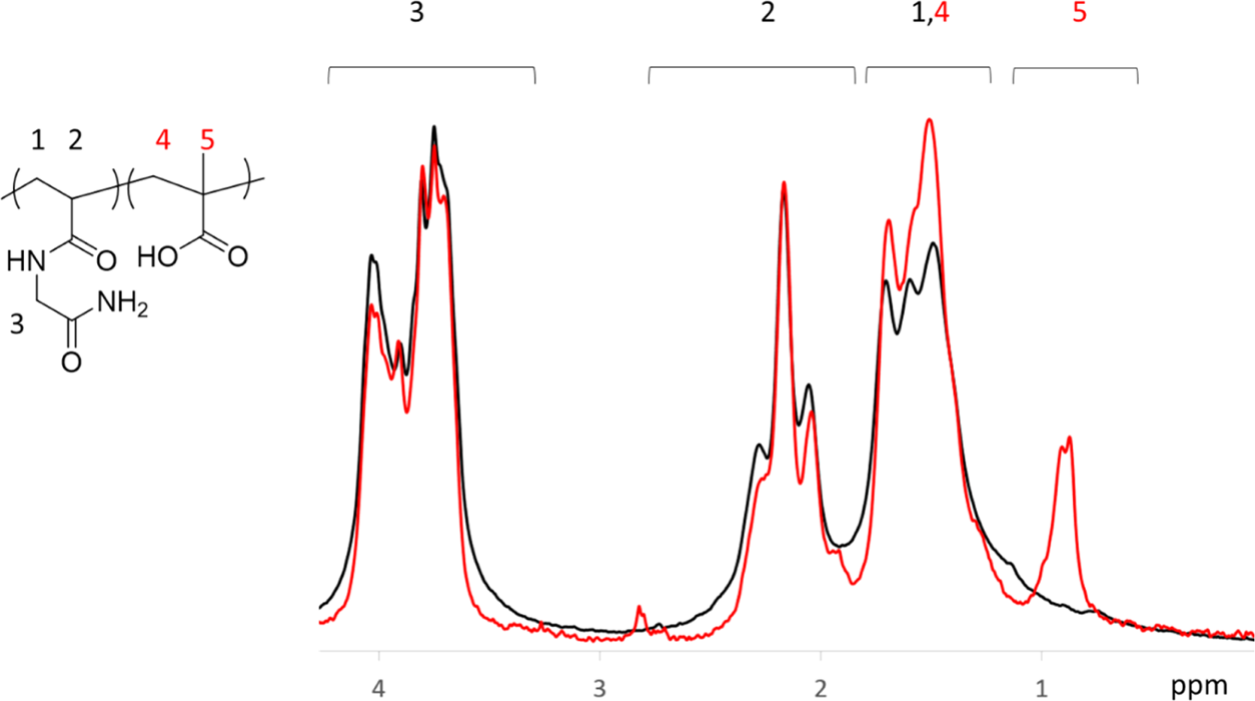
^1^H NMR spectra of P(NAGA) (black) and P(NAGA90–MAA10) (red) microgels at 23 °C.

A set of microgels varying the ratio of NAGA and MAA monomers was prepared by free radical polymerization. The polymerization was initiated by an APS/TEMED redox system in the presence of surfactant SDS and crosslinker methylenebis(acrylamide) (BIS) to achieve stabilized particles and a crosslinked structure. In order to stimulate the precipitation of the UCST-type microgels, the polymerizations were carried out below the expected VPTT of P(NAGA) but above the dissolution temperature of MAA. The ^1^H NMR signals from microgel dispersions were then used to calculate the monomer ratios of the copolymers, Figure 1. The copolymer feed ratio and the corresponding monomer ratios were found to correlate well, Table 1. Some residual lines were observed at around 2.8 ppm for the P(NAGA–MAA) microgels even after extensive purification. The signals are attributed to the adsorbed TEMED, as observed earlier for PNIPAM–TEMED systems.^32^

**Table 1:** Synthesis of Microgels

entry	feed ratio (NAGA/MAA)	yield^a^ (%)	copolymer composition^b^ (NAGA/MAA)
P(NAGA)	100:0		100:0
P(NAGA90–MAA10)	90:10	74	89:11
P(NAGA70–MAA30)	70:30	69	70:30
P(NAGA50–MAA50)	50:50	84	49:51
P(NAGA30–MAA70)	30:70	65	32:68

a After the purification steps.

b By NMR. The copolymer composition was taken as the ratio of the NAGA CH_2_ signals and the MAA methyl group signals.

**Figure 2:**
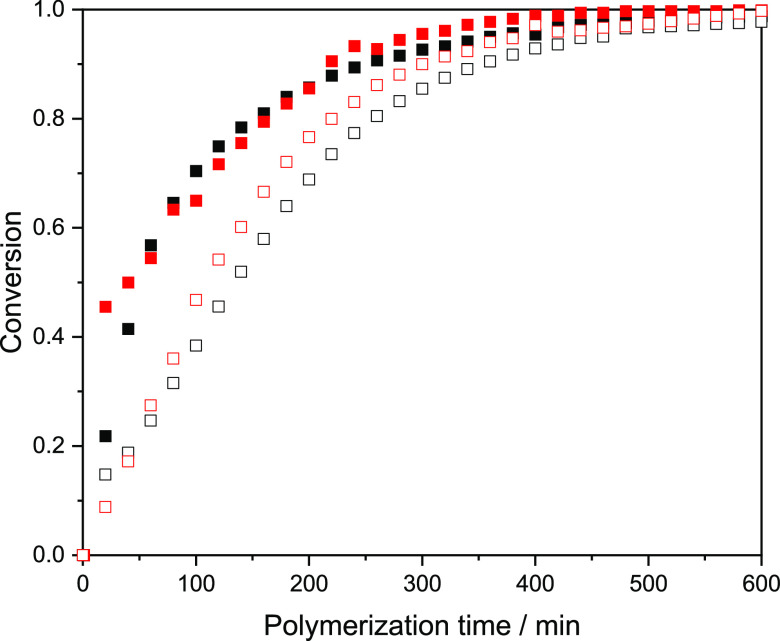
Conversion of P(NAGA70–MAA30) (NAGA black box solid, MAA red box solid) and P(NAGA50–MAA50) (NAGA black box, MAA red box) against reaction time.

The similar composition found in the samples does not necessarily confirm the copolymerization or formation of copolymer particles. In order to evaluate the randomness of the polymerizations, a kinetic study using the same reaction parameters as in the microgel syntheses was conducted by *in situ* NMR experiments. As shown in Figure 2, polymerizations containing either 30 or 50 mol % MAA show a similar rate of disappearance of the respective MAA or NAGA double bond signals. This indicates that the formed microgel networks do not likely have a blocky or a gradient structure but can instead be considered as having random monomer sequences. Similar results have been reported for the copolymerization of NAGA and MAA by RAFT polymerization at 70 °C.^30^

**Figure 3:**
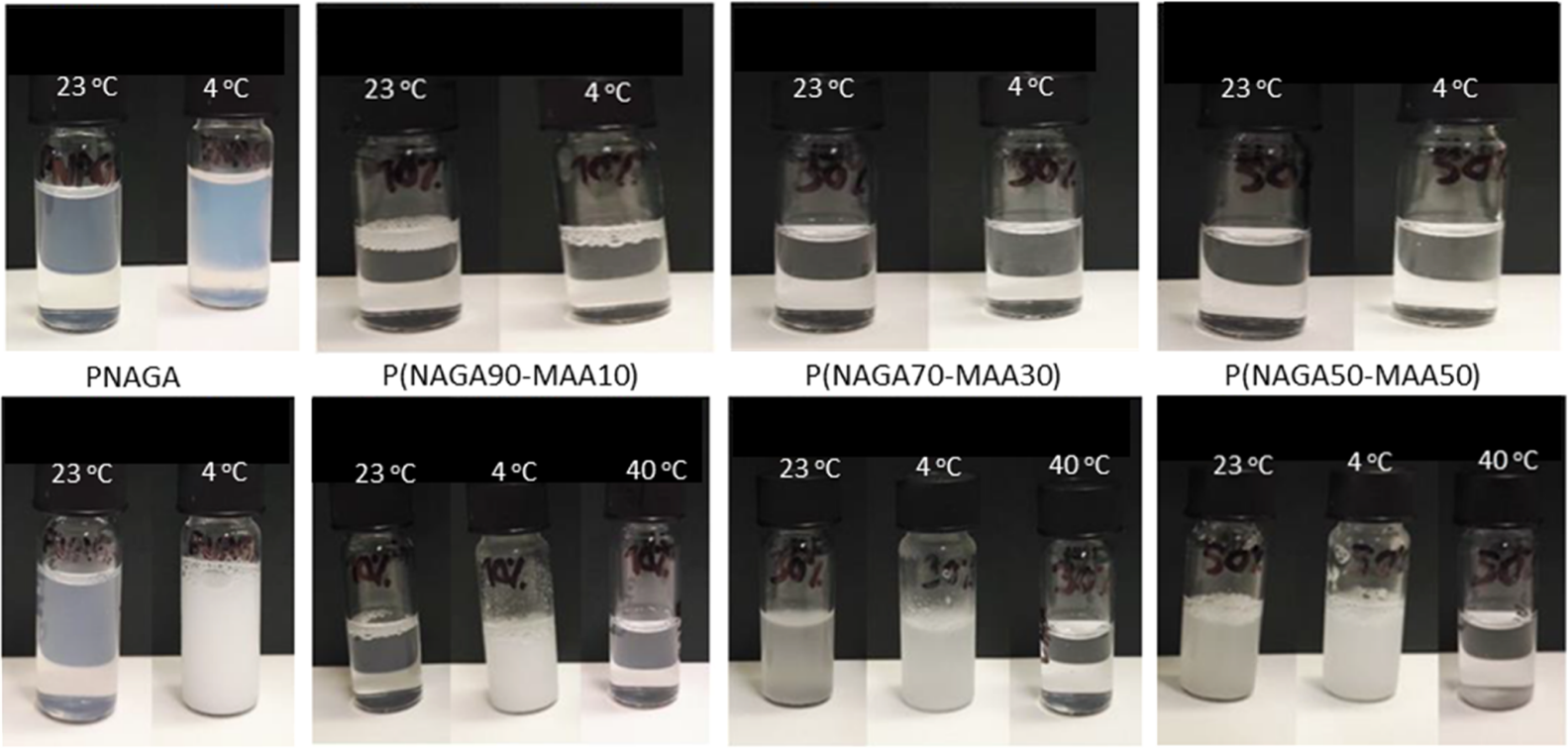
Top row: P(NAGA) and P(NAGA–MAA) dispersions at different temperatures without adjusting the pH. Bottom row: P(NAGA) and P(NAGA–MAA) at pH 3.

To determine the phase transition behavior of the microgels, they were first dispersed in neutral water at a 10 mg/mL concentration and observed at room temperature, Figure 3. All samples have a clear appearance, indicating the absence of large aggregates. When the temperature is lowered to 4 °C, the P(NAGA) homopolymer microgel shows visible clouding originating from the phase transition-induced aggregation. However, the MAA-containing samples stay clear upon cooling. It is known that even minute acrylic acidic impurities in linear homopolymer P(NAGA) are capable of suppressing its phase transition behavior in water due to the destabilization of the hydrogen bonds.^29^ Accordingly, the charged MAA units in the microgels affect the interactions between MAA and NAGA monomer units, resulting in disappearance of the phase transition behavior close to neutral pH.

The pH of the dispersions was lowered to pH 3 at room temperature. When the samples were cooled down to 4 °C, all samples become turbid and upon heating to 40 °C clear again. For P(NAGA) microgels, lowering the pH leads to more turbid dispersion at 4 °C compared to the one at neutral pH. This effect is related to the acrylic acid impurities mentioned above. At pH 3, these charges are screened and lead to increased turbidity. Lowering the pH below p*K*_a_ of MAA introduces the phase transition behavior observed for P(NAGA) and also for P(NAGA–MAA) microgels. However, at 23 °C, the samples with MAA content above 10% already show increased turbidity and the sample P(NAGA30–MAA70) did not clear at all upon heating to boiling, so it was omitted from further studies.

**Figure 4:**
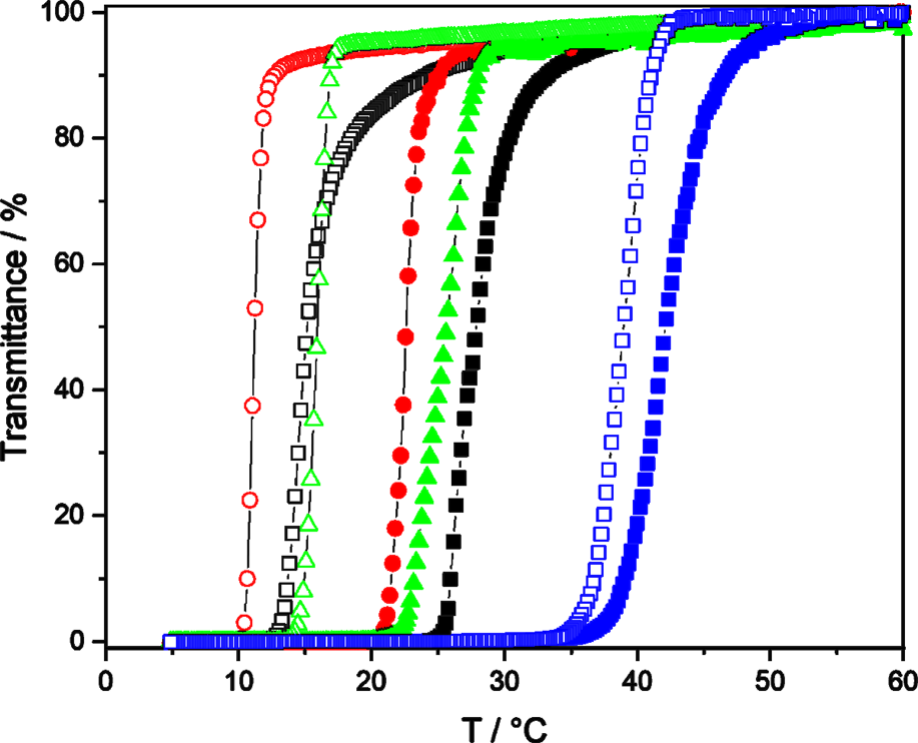
Turbidity curves measured for 10 mg/mL microgel dispersions at pH 3: P(NAGA) (black box solid, black box), P(NAGA90–MAA10) (red circle solid, red circle), P(NAGA70–MAA30) (green triangle up solid, green triangle up open), and P(NAGA50–MAA50) (blue box solid, blue box). Filled symbols heating, open cooling.

Turbidimetric characterization against temperature was performed at pH 3, Figure 4. A complex temperature dependence is observed for samples with different comonomer ratios, Table 2. Upon heating, the P(NAGA) microgel at pH 3 has an onset of phase transition, referred to as cloud point (*T*_c_), at 25 °C. When 10% MAA is incorporated, P(NAGA90–MAA10) microgels, *T*_c_ shifts to 21 °C and for P(NAGA70–MAA30) to 23 °C. A significant increase is observed for P(NAGA50–MAA50), where the onset is shifted to 36 °C and for the P(NAGA30–MAA70), which becomes completely insoluble. The drop in the phase transition temperature at MAA contents below 30 mol % and the increase at molar ratios above 30 mol % have been observed earlier for similar linear P(NAGA–MAA) copolymers at pH 4.^30^ It can be explained as a combined effect of inter/intramolecular hydrogen bonds between NAGA/MAA and the remaining ionization of the MAA units at a pH close to the p*K*_a_ of MAA. Thus, at a low MAA ratio, the randomly distributed, relatively few MAA units disturb the hydrogen bonds between NAGA units without yet being capable of forming new as strong bonds with either NAGA units or themselves. With increasing MAA content, the bonding strengthens, but turbidimetry alone does not reveal the mechanism.

**Table 2:** Phase Transition Behavior of the Microgels at pH 3

entry	*D*_h_ at 15/25/40 °C^a^ [nm]	onset heating [°C]	onset cooling [°C]	hysteresis [°C]
P(NAGA)	73/83/80^b^	25	15	11
P(NAGA90–MAA10)	44/50/63	21	11	11
P(NAGA70–MAA30)	47/57/80	23	16	9
P(NAGA50–MAA50)	404/240/116	36	38	4

a By DLS.

b Measured at neutral pH.

Analogous complex hydrogen bonding interactions between amide and carboxylic acid groups have been reported also for LCST-type systems. For PNIPAM–MAA microgels studied at pH 3.4, the increase of MAA content lowered the transition temperature and at MAA contents above 50 mol % lead to collapsed microgels at all studied temperatures.^21^ For poly(*N*-isopropylacrylamide–acrylic acid) PNIPAM–AA copolymers, the incorporation of AA units in random leads to increase of the phase transition temperature at low pH, yet when the AA units are polymerized to segments, the temperature decreases.^22^

Similar to linear P(NAGA) and copolymers,^29,30^ there is a notable hysteresis in the phase transition temperature of the microgels upon heating and cooling, manifesting that the phase transition is a kinetically controlled phenomenon. The hysteresis decreases significantly for only the P(NAGA50–MAA50) sample. As P(NAGA50–MAA50) also has the highest UCST-type phase transition temperature, the effect is likely related to generally stronger interactions present in the system, leading to the elevated phase transition temperature and faster kinetics.

When the microgel samples are studied at a more dilute concentration (1 mg/mL) by DLS, Table 2, the copolymer microgels with an MAA content of 10 and 30% swell upon heating, while the 50% microgels appear to shrink. However, unlike in the turbidimetric experiments, the change in dimensions is gradual over the studied temperature range, Figure S1. The behavior of the 10 and 30% microgels is similar as observed earlier in our studies for P(NAGA) microgels^26^ and shows that at dilute concentrations, these nonaggregated microgels undergo a gradual volume change both upon cooling and heating instead of a clear phase transition. Moreover, the sample with the highest MAA content studied, P(NAGA50–MAA50), shows at low temperatures much larger particles that decrease in size upon heating but start to grow after a minimum is reached and upon further heating. When observing the respective data of P(NAGA50–MAA50) in Figures 3 and S1, it is obvious that the behavior originates from de-aggregation of the particles upon heating, followed by swelling after the heating-induced deaggregation.

The UCST-type thermal behavior of P(NAGA) results from the change in hydrogen bonding between the amide groups. Upon heating, the amide–amide hydrogen bonds are cleaved, which is an endothermic process. However, the simultaneously forming amide–water hydrogen bonds produce an exothermic signal. Therefore, the observation of the enthalpy changes in case of P(NAGA) is complicated.^29^ So far, to our knowledge, two reports on the calorimetry of P(NAGA) exist. Agarwal *et al.*(29) reported that a carefully purified, nearly acrylic acid-free, linear P(NAGA) 10 mg/mL solution in water had an endothermic transition between 5 and 25 °C. We reported for a P(NAGA) microgel in PBS a broad endothermic transition between 30 and 60 °C.^27^

**Figure 5:**
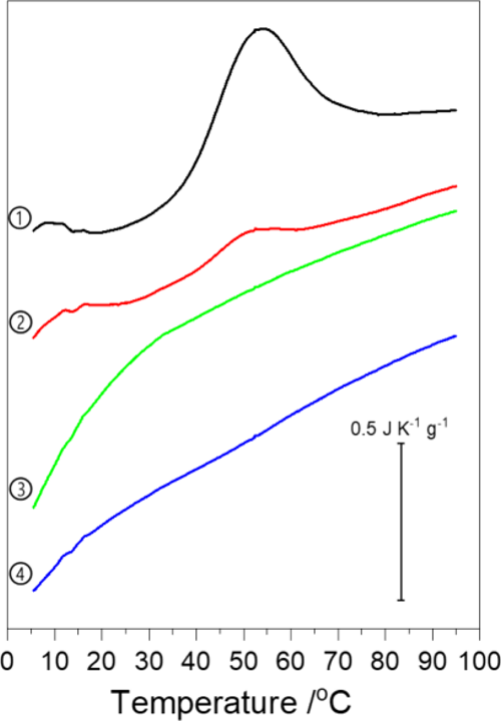
Microcalorimetry scans in water at pH 3. 1. P(NAGA) 2. P(NAGA90–MAA10) 3. P(NAGA70–MAA30) 4. P(NAGA50–MAA50).

Concentration-normalized microcalorimetric scans of the microgel dispersions are shown in Figures 5 and S2. P(NAGA) microgel shows two broad endothermic transitions around 5–20 and 30–80 °C both at neutral pH and at pH 3. Similar results are obtained in D_2_O and in water. Lowering the pH shifts the maxima of heat flows to slightly higher temperatures and amplifies the enthalpic change especially in the case of the P(NAGA) microgel. Both effects likely follow from the screening of the residual carboxylate impurities’ charges at pH 3, which promotes increased hydrogen bonding. In order to compare with earlier literature, a linear P(NAGA) reference polymer was analyzed under the same conditions, Figure S3. In this case, the higher temperature transition is divided in two, and the polymer shows instead of two, three separate endothermic transitions.

The enthalpies of the transitions of the different P(NAGA) samples are shown in Table 3. These values should be considered qualitative due to the large error following from the small and broad signals. Nevertheless, the enthalpy of the low-temperature transitions are generally of the same order of magnitude as the value reported earlier (0.7 J/g).^29^ The endothermic signal of P(NAGA) samples observed at higher temperatures has apparently an order of magnitude higher enthalpy. Our hypothesis for the observation of several maxima is that both endo- and exothermic processes are taking place and lead to the complicated signals. This effect is pronounced, for example, with the P(NAGA) microgel at pH3 in D_2_O, Figure S2, where a clear exothermic deviation from the baseline can be observed between 20 and 30 °C. A similar effect can also explain the three maxima observed in the linear P(NAGA) thermograms.

**Table 3:** Microcalorimetry of P(NAGA) Microgels and Linear P(NAGA)

entry	peak	max heat flow [°C]	enthalpy [J/g]	total enthalpy [J/g]
P(NAGA) in D_2_O	1	12	2.5	
	2	40	3.2	5.7
P(NAGA) pH 3 in D_2_O	1	14	1.3	
	2	45	10.6	11.9
P(NAGA) pH 3 in water	1	10	0.2	
	2	53	11.9	12.1
linear P(NAGA) in D_2_O	1	10	0.6	
	2	45	7.9	
	3	73	2.3	10.8
linear P(NAGA) pH 3 in D_2_O	1	12	1.3	
	2	45	5.6	
	3	73	2.4	9.3
linear P(NAGA) pH 3 in water	1	12	0.6	
	2	40	5.3	
	3	69	3.8	9.6

For the copolymer samples studied at pH 3, the microgel with the smallest amount of MAA, P(NAGA90–MAA10), shows both endothermic signals observed in the case of the P(NAGA) microgel but suppressed and shifted to a lower temperature. For P(NAGA70–MAA30), both are still present in D_2_O but in water only a broad endothermic deviation from the baseline can be observed. For the P(NAGA50–MAA50), the signal is further suppressed. The monotonic decrease of the endothermic signals with the increase of the MAA content suggests that the calorimetry primarily detects the breaking of hydrogen bonds between NAGA units. The change of interactions between the NAGA and MAA units or between the MAA units is thus not detected, meaning that the processes are either athermal, compensated by the formation of new hydrogen bonds or below the sensitivity of the calorimeter. However, as observed by turbidimetry, these processes eventually lead to macroscopic phase change.

**Figure 6:**
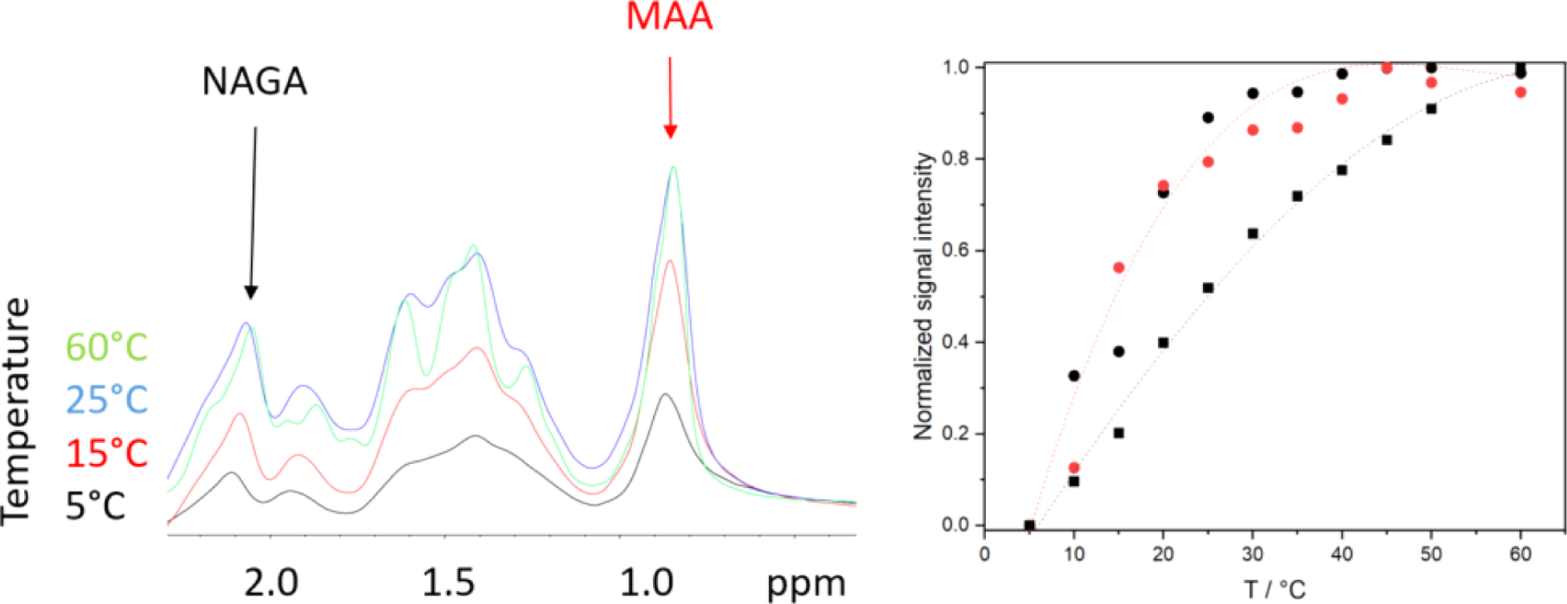
^1^H NMR spectra of P(NAGA70–MAA30) at pH 3 at 5, 15, 25, and 60 °C. On the right ,normalized signal intensity for P(NAGA) (black box solid) and P(NAGA70–MAA30) NAGA (black circle solid) and MAA (red circle solid) signals. Lines are to guide the eye.

When the copolymer microgels are dispersed in neutral D_2_O and studied by NMR at different temperatures, no signal intensity changes are observed, which is in line with the visual observations, Figure 3. When the samples are studied at pH 3, the signals below 10 °C are suppressed for both NAGA and MAA repeating units, Figure 6, indicating slow polymer dynamics due to inter- and intrapolymer interactions as observed earlier for P(NAGA) microgels.^26,27^ Upon heating, the NMR signals intensify. For the P(NAGA) microgel at pH 3, Figure 6, the increase is continuous up to the studied temperature of 60 °C. For the copolymer microgels, the increase in the signal intensities is steep at temperatures of 5–35 °C after which the signals level off. This temperature range fits well between the apparently contracting turbidimetry and microcalorimetry results. Importantly, for most of the copolymer microgels, the signals from NAGA and MAA intensify similarly regardless of the NAGA–MAA ratio, Figure S4, indicating that both types of repeating units are simultaneously involved in the phase separation process. This is in agreement with the polymerization kinetics results, supporting the conclusion that the copolymers have a random distribution of different repeating units and do not form, for example, homopolymer microgel particles.

The transition temperature upon heating observed with turbidimetry, Table 2, and the endotherm maxima by calorimetry, Table 3, differ from each other and the NMR results for all of the samples studied. It is clear that the experimental methods probe different aspects of the phase transition process. The dynamics of the polymer chains are slowed down at low temperatures, evidenced by the broadened NMR signals. This is due to inter- and intramolecular hydrogen bonds between first the NAGA amide groups but also the repeating units of NAGA and uncharged MAA. These hydrogen bonds break upon heating of the samples, and this takes place even at relatively low temperatures, possibly throughout the studied temperature range. The endothermic phase transition at 5–15 °C, corresponding to the temperature where the signal intensity most drastically changes in NMR measurements. However, at these temperatures, no visual changes are observed in the dispersions, indicating that the low-temperature processes occur within the crosslinked networks but do not change the state of aggregation of the microgels. The microcalorimetric measurements show another endothermic process of larger magnitude at higher temperatures. At these temperatures, the NMR line intensities are already saturated for the copolymer microgels. This would indicate that this process is related to the overall phase transition-induced (de)aggregation on a macroscopic level, which is then observed by turbidimetric and scattering experiments. A similar difference between the characterization methods has been noted for LCST-type aqueous solutions of hydrophobically modified poly(2-isopropyl-2-oxazolines)^33^ and azopyridine-modified PNIPAM.^34^ In these cases, the association into larger objects upon heating is readily detected by turbidimetry, but the detection of phase transition by DSC or NMR is only observed at higher temperatures. Though the LCST-type transition is opposite and the enthalpy changes are much more clearly observable by calorimetry, the processes bear similarities.

### AgNP Loading and Catalysis

**Figure 7:**
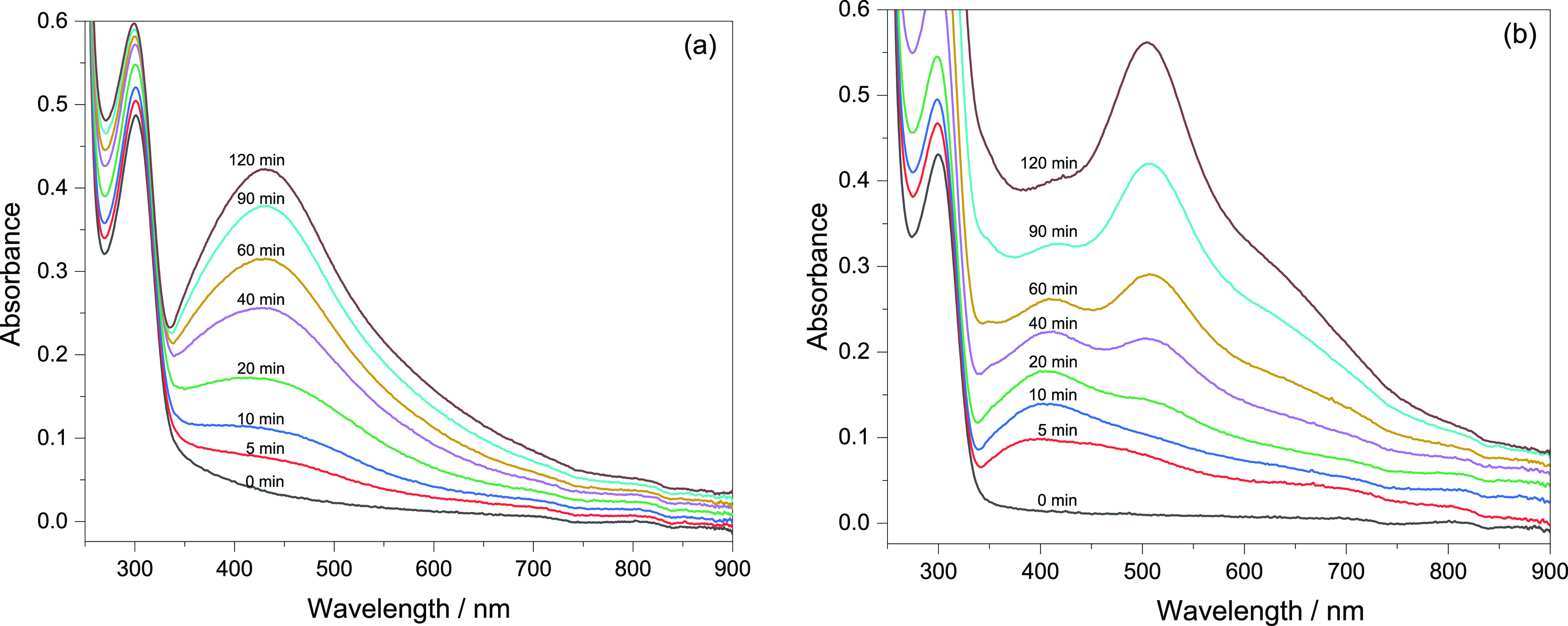
UV-spectra upon irradiation of the (a) P(NAGA) and (b) P(NAGA70–MAA30) microgels in AgNO_3_ dispersions.

When considering the use of these stimuli-responsive systems as nanocatalysts, the ionizable carboxylic acid groups of MAA units are attractive for reduction of metal cations to a small size, even as nanoclusters.^15,35,36^ In the present case, the loading of AgNPs was done by UV-irradiation with the aim to make smaller NPs compared to chemically reduced AgNPs and thus maximize their surface area.^31^ The formation of AgNPs by 365 nm irradiation was followed by recording the UV-spectra, Figure 7. For the P(NAGA) microgel, the intensity of the surface plasmon resonance band of AgNPs centered at ∼430 nm increases throughout the irradiation time. The constant band position at 430 nm indicates that the formed particles have dimensions larger than *ca.* 2–3 nm.^37^ When the same experiment is followed for the P(NAGA70–MAA30), it can be observed that first a broad band located at 405 nm starts to appear, which then is overlapped with a band appearing at 505 nm as well as development of a shoulder appearing at around 360 nm. According to an earlier report on AgNP-loaded poly(*N*-isopropylacrylamide–acrylic acid–hydroxyethyl acrylate) microgels^38^ prepared by the UV-irradiation method, the shoulder at 330–360 nm and the peak at 490–520 nm arise from absorption by clusters of silver, Ag_*n*_^+^, where n varies from 4 to 9.

**Figure 8:**
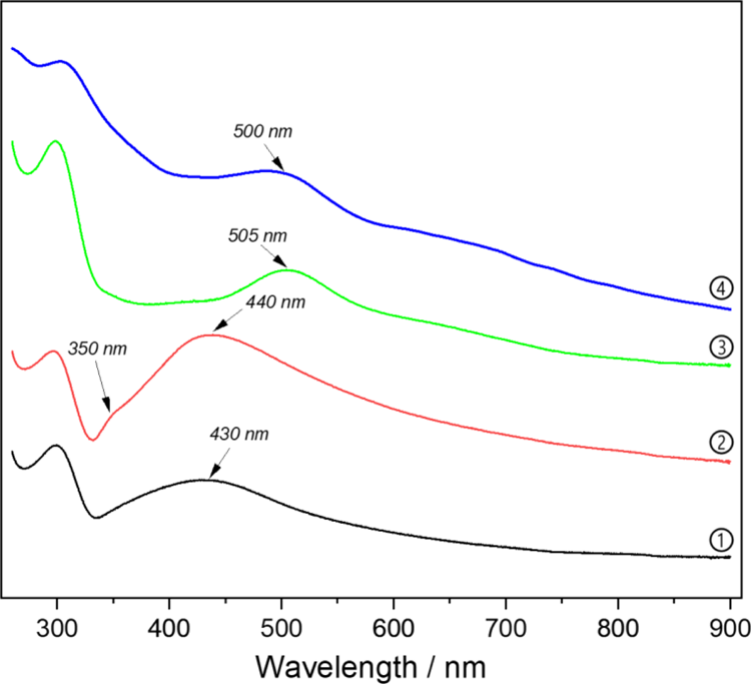
UV-spectra of the AgNP–P(NAGA–MAA) microgels after dialysis. 1. AgNP–P(NAGA) 2. AgNP–P(NAGA90–MAA10) 3. AgNP–P(NAGA70–MAA30) 4. AgNP–P(NAGA50–MAA50).

When the UV-spectra of the series of microgel samples are compared after dialysis, it can be observed that with increasing microgel MAA content, the plasmon bands are red-shifted, Figure 8. In addition, the MAA-containing samples show plasmon bands at around 430 and 505 nm, which were already observed during the irradiation and indicate the presence of silver nanoclusters.

**Figure 9:**
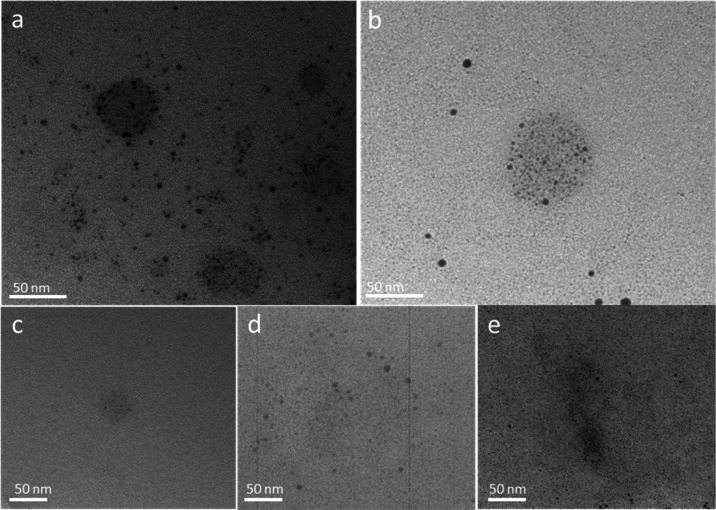
TEM micrographs of AgNP-containing microgels. From left to right: Ag–P(NAGA) reduced with NaBH4, P(NAGA) UV, P(NAGA90–MAA10), P(NAGA70–MAA30), and P(NAGA50–MAA50).

TEM micrographs of the resulting microgel particles after Ag loading are shown in Figure 9. As seen in Figure 9a, the chemical reduction with NaBH_4_ produces larger nanoparticles, similarly as reported earlier for P(NAGA) microgels.^26^ When comparing this to the UV-reduced AgNP–P(NAGA) and AgNP–P(NAGA90–MAA10) microgels, in those cases, the silver particles appear more finely dispersed, especially in the case of the MAA-containing sample. For the samples with a higher MAA content, AgNP–P(NAGA70–MAA30) and AgNP–P(NAGA50–MAA50), the images show some large, clustered AgNPs, but more importantly the whole background appears stained and consisting of AgNPs or Ag nanoclusters. This result is in accordance with the TGA results, Table 4, as the higher MAA content leads to a higher overall silver content.

**Table 4:** AgNP Content and Catalytic Efficiency of Microgels

entry	Ag-content [m %]	apparent rate constant [s^–1^]	Ag mass-corrected rate constant [s^–1^ mg^–1^]
AgNP–P(NAGA)^a^	8.0	0.007	5
AgNP–P(NAGA)^b^		0.008	
AgNP–P(NAGA90–MAA10)	15.6	0.037	4
AgNP–P(NAGA70–MAA30)	18.3	0.262	25
AgNP–P(NAGA50–MAA50)	17.3	0.356	42

a Ag reduced with NaBH_4_.

b Ag content could not be determined.

**Figure 10:**
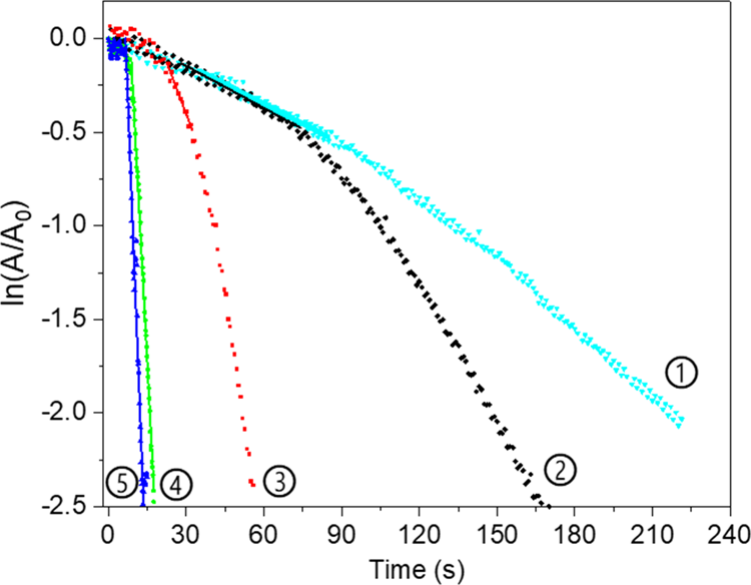
Kinetics of 4-NP catalysis monitored by UV. 1 Chemically reduced AgNP–P(NAGA), 2 AgNP–P(NAGA), 3 AgNP–P(NAGA90–MAA10), 4 AgNP–P(NAGA70–MAA30), and 5 AgNP–P(NAGA50–MAA50). The lines indicate the fits used to calculate the rate constants.

The kinetics of 4-nitrophenol reduction were studied at 30 °C by UV, Figure S5. At this temperature, all the studied microgels are swollen, allowing their comparison. The kinetic plots, Figure 10, show that the chemically reduced AgNP–P(NAGA) has a roughly similar induction time before catalysis begins compared with the P(NAGA) microgel with silver reduced under UV light. The result indicates that the rate of diffusion of the reactants/products to the microgels is similar. However, the catalysis rate is higher in the case of photocatalyzed AgNPs. Based on the TEM image, this results from the smaller size of the AgNPs and correspondingly their larger surface area.

With increasing MAA content, the induction time is shortened significantly and the rate of catalysis increases. Under neutral conditions, the MAA units cause the microgels to swell and allow faster diffusion inside the microgels, resulting in almost immediate catalysis in the case of 30 and 50% MAA-containing AgNP microgels. According to TGA, the more MAA-containing microgels have also higher m % of AgNPs and the kinetics data was normalized with respect to the Ag mass. The mass-normalized data show that the catalysis efficiency increases with increasing MAA content. As the surfaces of the Ag-particles are responsible for the catalytic transformation, the smaller the Ag-particle size, the faster the reaction kinetics. Unfortunately, the sizes of the fine Ag-particles cannot be quantified from the TEM images.

## Conclusions

Dual thermo- and pH-sensitive microgels based on P(NAGA–MAA) were prepared and their phase transition behavior and Ag-nanoparticle catalyst encapsulation and catalysis efficiency were investigated. Copolymerization of the NAGA and MAA monomers produced microgels with monomer ratios close to their feed ratio. The sizes of the microgels were of the order 60–120 nm in the unaggregated swollen state and they show a UCST phase transition.

The P(NAGA) homopolymer microgel showed phase transitions in both neutral and acidic pH, manifested in reduced polymer dynamics at low temperatures, clouding at lower temperature, and clearing upon heating. The MAA-containing P(NAGA–MAA) copolymer microgels did not show phase transitions at neutral pH, as above the p*K*_a_ of MAA, the hydrogen bonding interactions between NAGA and MAA units are weakened. At pH 3, all copolymer microgels show a UCST-type temperature-sensitive behavior, clouding at lower temperature and clearing upon heating. However, the cloud point (*T*_c_) showed a complex dependence of the comonomer ratio, the *T*_c_ first decreasing upon addition of MAA up to 10 mol % and then increasing with higher MAA contents. Microcalorimetric investigations revealed two endothermic processes occurring during the heating of P(NAGA) microgels. For copolymer microgels, the transitions occurred at lower temperatures and the enthalpies decreased with increasing MAA content. Turbidimetry, calorimetry, and NMR investigations reveal different aspects of the phase transition process and yield different temperatures.

The microgels were loaded by AgNPs by UV-irradiation of the silver nitrate solution. The photoreduction produced smaller AgNPs than chemical reduction by NaBH_4_. The amount of the AgNPs increased with increasing microgel MAA molar ratio and the size of the AgNPs decreased at the same time. Catalysis tests using 4-nitrophenol reduction as a model reaction showed fastest reaction kinetics for the microgels with the highest MAA molar ratio due to the high loading and surface area of the AgNPs.

## Supporting Information Available

Comparison of microgels’ hydrodynamic diameters between different light scattering instruments; microcalorimetric scans of microgels in D_2_O and linear P(NAGA) in water and D_2_O; NMR signal intensities at different temperatures for microgel dispersions; and UV–vis absorption spectra of the 4-nitrophenol solution before and after catalysis (PDF)
