# Supplementary material for: Phase Transition Behavior and Catalytic Activity of Poly(N-acryloylglycinamide-co-methacrylic acid) Microgels
Source: Langmuir. 2021 Feb 17;37(8):2639–48. doi: 10.1021/acs.langmuir.0c03264 (PMC8026100; doi:10.1021/acs.langmuir.0c03264)
Supplement: Supplementary file 1 — la0c03264_si_001.pdf [file la0c03264_si_001.pdf]

## Supporting information for

### Phase Transition Behavior and Catalytic Activity of Poly(N-Acryloylglycinamide-co-Methacrylic Acid) Microgels

Dong Yang, Heli Eronen, Heikki Tenhu, Sami Hietala*

Department of Chemistry, University of Helsinki, P. O. Box 55, FIN-00014 HU, Finland.

* E-mail: sami.hietala@helsinki.fi

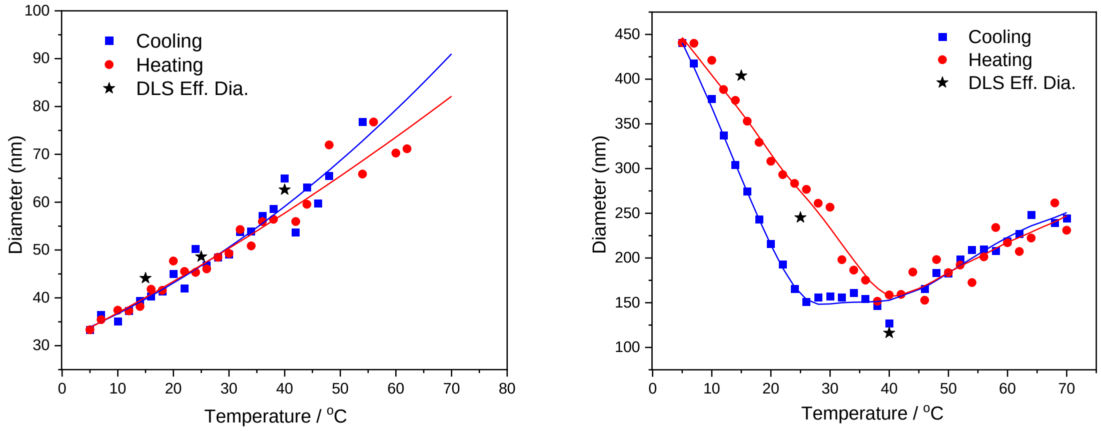


*Figure S1. DLS and zetasizer data compared for P(NAGA90-MAA10) (left) and P(NAGA50-MAA50) (right).*

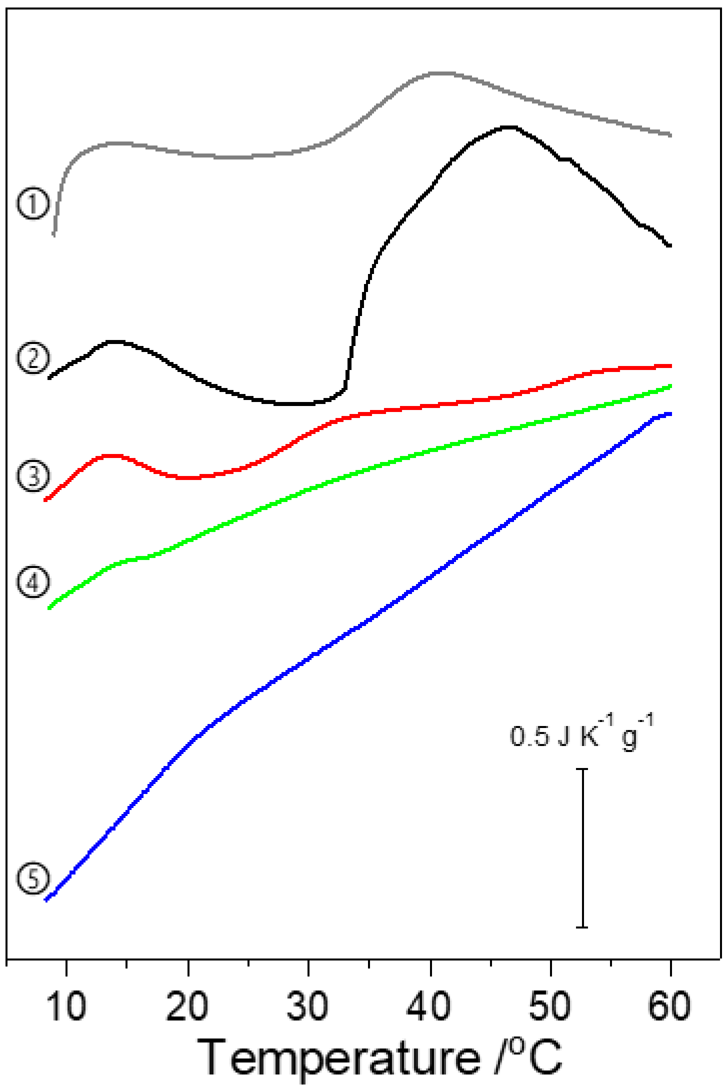


*Figure S2. Microcalorimetric scans of microgels in D2O. 1. PNAGA in D2O, 2. PNAGA pH=3, 3. P(NAGA90-MAA10) pH=3, 4. P(NAGA70-MAA30) pH=3, 5. P(NAGA50-MAA50) pH=3.*

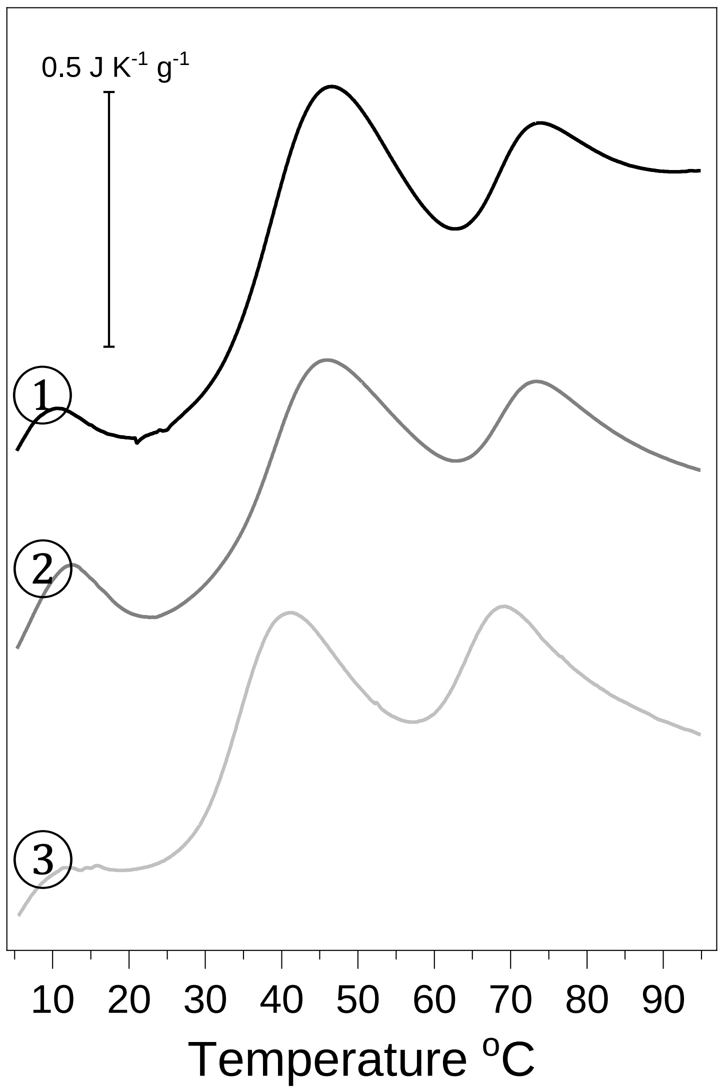


*Figure S3. Microcalorimetric scans of linear PNAGA. 1. D2O, 2. D2O pH=3, 3. water pH=3.*

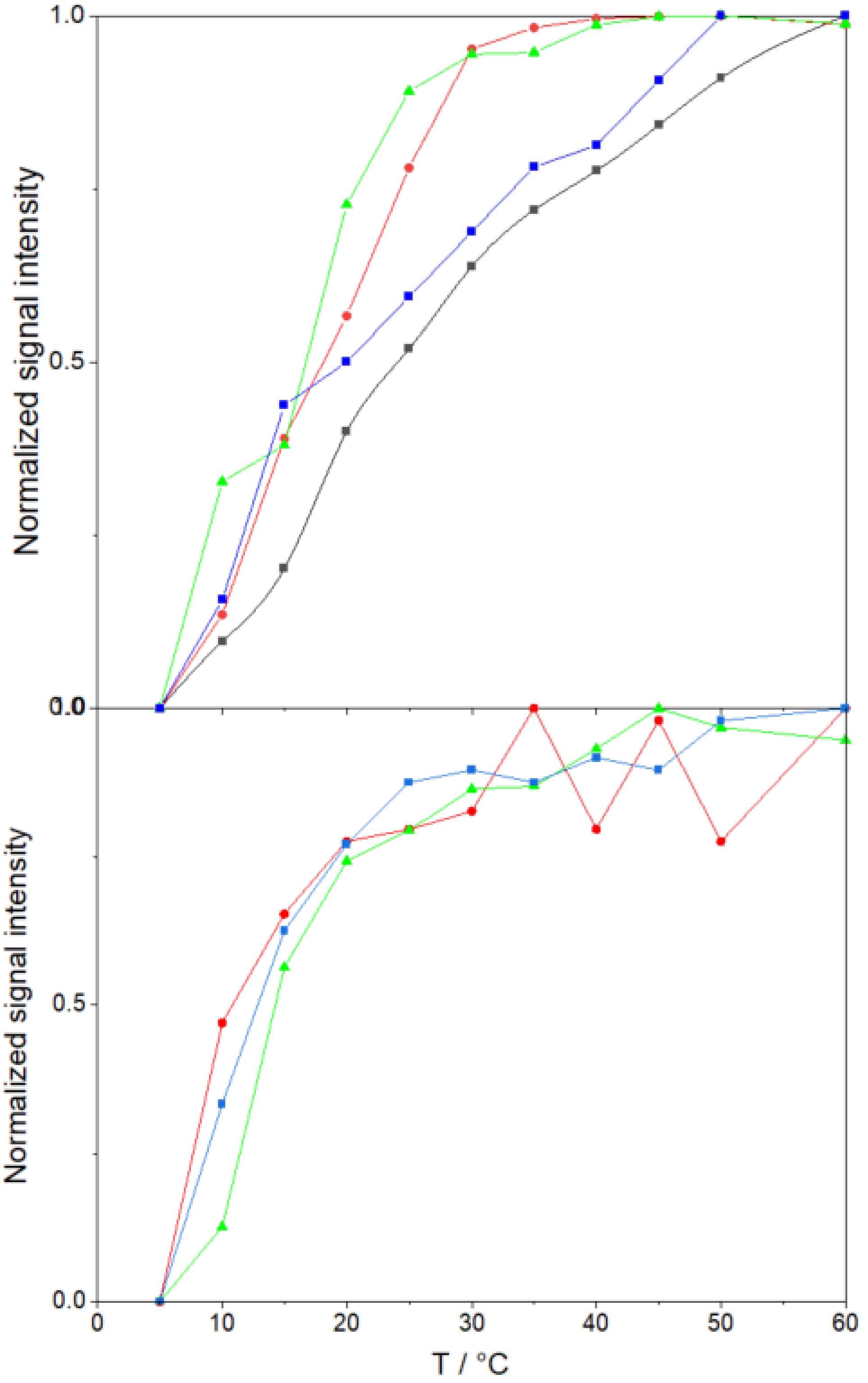


*Figure S4. Variable temperature NMR signal intensities for different microgel dispersions. Top panel: NAGA signal intensity (2.1 ppm); bottom panel: MAA signal intensity (0.9 ppm). PNAGA (■), P(NAGA90-MAA10) (●), P(NAGA70-MAA30) (▲) and P(NAGA50-MAA50) (■).*

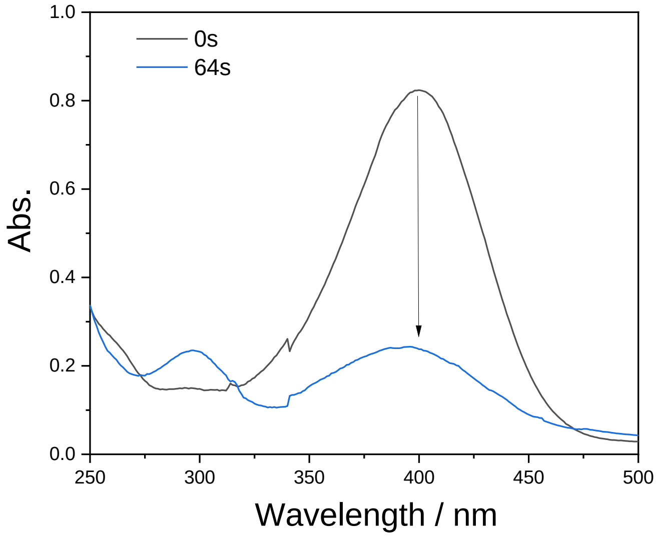


*Figure S5. UV-VIS absorption spectra of 4-nitrophenol solution before reduction and 64 seconds after addition of 13 ul of AgNP-P(NAGA50-MAA50) dispersion as the catalyst.*
